# Molecular couplings and energy exchange between DNA and water mapped by femtosecond infrared spectroscopy of backbone vibrations

**DOI:** 10.1063/1.4980075

**Published:** 2017-04-07

**Authors:** Yingliang Liu, Biswajit Guchhait, Torsten Siebert, Benjamin P. Fingerhut, Thomas Elsaesser

**Affiliations:** Max-Born-Institut für Nichtlineare Optik und Kurzzeitspektroskopie, 12489 Berlin, Germany

## Abstract

Molecular couplings between DNA and water together with the accompanying processes of energy exchange are mapped via the ultrafast response of DNA backbone vibrations after OH stretch excitation of the water shell. Native salmon testes DNA is studied in femtosecond pump-probe experiments under conditions of full hydration and at a reduced hydration level with two water layers around the double helix. Independent of their local hydration patterns, all backbone vibrations in the frequency range from 940 to 1120 cm^–1^ display a quasi-instantaneous reshaping of the spectral envelopes of their fundamental absorption bands upon excitation of the water shell. The subsequent reshaping kinetics encompass a one-picosecond component, reflecting the formation of a hot ground state of the water shell, and a slower contribution on a time scale of tens of picoseconds. Such results are benchmarked by measurements with resonant excitation of the backbone modes, resulting in distinctly different absorption changes. We assign the fast changes of DNA absorption after OH stretch excitation to structural changes in the water shell which couple to DNA through the local electric fields. The second slower process is attributed to a flow of excess energy from the water shell into DNA, establishing a common heated ground state in the molecular ensemble. This interpretation is supported by theoretical calculations of the electric fields exerted by the water shell at different temperatures.

## I. INTRODUCTION

The structure and function of biomolecules are intrinsically coupled to their aqueous environment.^1^ Water forms a hydration shell which interacts with a biomolecular system through local hydrogen bonds, short- and intermediate-range Coulomb forces from the dipolar water molecules, and hydrophobic forces. In the case of DNA, the hydration pattern at the surface of the double helix is highly inhomogeneous with the rather rigid so-called spine of water molecules in the minor groove and a preferred hydration of the ionic phosphate groups of the backbone.^2–6^ Each phosphate group of the B-DNA helix, the structure prevalent under physiological conditions, is hydrated by up to 6 water molecules forming hydrogen bonds with the free ${\text{PO}}_{2}^{-}$ oxygens.^7^ Electric forces at the DNA surface have been shown to mainly originate from water molecules in the first two hydration layers.^8–10^ The time averaged electric field amplitude reaches values up to some 90 MV/cm.

The thermal motions of water molecules in the hydration shell are connected with structural fluctuations, e.g., librational motions, in the femtosecond time domain. Such fluctuations are moderately slowed down by a factor of 3–5 compared to neat bulk water.^11–15^ Hydrogen bond breaking and reformation occur both between water molecules in the hydration shell and for water molecules forming H-bonds with the biomolecule. The underlying process is the molecular jump mechanism which consists in large angular jumps of water molecules between the original and the new hydrogen bonding geometry.^16^ For hydrogen bonds between water and biomolecular units, the time scale depends on the hydrogen bond strength and the topology of the biomolecule's surface, again leading to a moderate slowing down compared to the few-picosecond dynamics in the bulk outer part of the hydration shell. An exception is the water molecules in the minor groove which form long-lived hydrogen bonds.^13^

The structural fluctuations give rise to (ultra)fast fluctuations of the electric field acting on the DNA backbone with a fluctuation amplitude on the order of ±25 MV/cm.^10^ The electric interactions between the water shell and the DNA helix have recently been mapped by ultrafast two-dimensional (2D) infrared spectroscopy of backbone vibrations which serve as molecular probes at the water-DNA interface.^9^ The lineshape of the 2D spectra has allowed for extracting frequency-time correlation functions for the different vibrations. The correlation functions of all modes consist of an initial 300 fs decay representing the time scale of electric field fluctuations and a long-lived component accounting for a quasi-static inhomogeneous broadening due to structural disorder.

Beyond the electric interactions, there are processes of energy exchange and transfer between DNA and its water shell. They are highly relevant for redistributing excess energy and establishing a thermodynamic equilibrium state after the decay of electronic and/or vibrational excitations. An efficient management of excess energy is essential for the biomolecule's structural integrity and stability against thermal decomposition. While picosecond time scales of energy transfer have been established for molecular systems in the liquid phase,^17^ energy exchange and transfer between biomolecules and water are barely understood. Major issues are the following:

• What are the mechanisms, time scales, and rates of energy transfer between DNA and its water shell and vice versa?
• How do energy transfer kinetics between DNA and water compare to vibrational relaxation and equilibration within DNA and within the water shell, i.e., does energy transfer occur between equilibrated subsystems?
• Which degrees of freedom are involved in energy transfer, i.e., how local or delocalized are the motions involved and over what dimensions do the structural units extend that must be considered in describing the transfer rates? Is there a distribution of transfer rates which originates from the structural heterogeneity of the hydration shell?
• How can energy transfer processes be probed in experiments on femto- to picosecond time scales?

In this article, we report a comprehensive study of DNA-water couplings and energy exchange by ultrafast vibrational spectroscopy. Native salmon testes DNA containing some 2000 base pairs is studied under conditions of full hydration and at a reduced hydration level of roughly two closed water layers around the double helix. We consider a scenario in which a femtosecond mid-infrared pump pulse excites the water shell via the OH stretch absorption band. The DNA response is probed in a time-resolved way by mapping transient absorption changes of backbone modes which are sensitive probes located at the DNA-water interface. The probe pulses cover a frequency range between 940 and 1120 cm^−1^. The experiments cover more than two orders of magnitude in time from less than 1 up to 100 ps, in order to get insight into slower picosecond processes. The behavior observed after OH stretch excitation is benchmarked by measurements in which the backbone modes are excited and probed resonantly.

Our results reveal a quasi-instantaneous response of vibrational absorption on all backbone modes, independent of their local hydration geometries. This initial response is followed by kinetics on time scales of a few and a few tens of picoseconds. The resulting long-lived absorption changes reveal the formation of a common hot ground state of DNA and water. The few-picosecond kinetics are assigned to water-DNA couplings mediated via the electric field the hydration shell exerts on the backbone while the time evolution in the regime of tens of picoseconds reflects a flow of excess energy from the water shell into the DNA helix.

## II. METHODS

Femtosecond pump and probe pulses independently tunable in the mid-infrared are generated in two home-built optical parametric amplifiers (OPA) pumped by the output of an amplified Ti:sapphire laser system.^18^ In each OPA, a small part of the 805 nm pump pulses at a 1 kHz repetition rate generates a supercontinuum seed in a 1 mm thick sapphire plate, serving as the signal frequency input for parametric amplification in a barium beta-borate (BBO) crystal which is used in a double-pass geometry. Subsequent difference frequency mixing of signal and idler pulses in a 0.75 mm thick AgGaS_2_ crystal provides pulses centered at 3450 cm^−1^ with an energy of 1.6 *μ*J, while difference frequency generation in a 0.75 mm thick GaSe crystal (z-cut, *θ* = 34.0°–36.5°) gives pulses tunable in the frequency range from 900 to 1200 cm^−1^. The pulse-to-pulse stability in energy is better than 0.5%. Pump-probe measurements are performed with pump pulses at 3450 cm^−1^, 1095 cm^−1^, and 1005 cm^−1^ while the probe pulses are centered at 1095 and 1005 cm^−1^ with a bandwidth of 150 cm^−1^. The intensity ratio between the pump and the probe is 50:1 and the time resolution is on the order of 100 fs. All measurements are performed with parallel linear polarizations of the pump and the probe.

Pump and probe pulses interact with the DNA sample in a noncollinear geometry, using an off-axis parabolic mirror for focusing on the sample and a second mirror to image the transmitted probe light onto the detection system. The spot diameter on the sample is approximately 100 *μ*m. In addition, a reference probe beam is introduced which travels through an unexcited part of the sample. The probe and reference beams transmitted through the sample are spectrally dispersed and detected by a 2 × 64-element HgCdTe detector array (spectral resolution 2 cm^−1^). Normalizing the transmitted probe to the reference intensity allows for measuring absorption changes of the sample down to ΔA=0.03 mOD. The fraction of water molecules excited in the pumped sample volume is 3%–4%.

Salmon testes DNA (Aldrich), which contains approximately 2000 base pairs (41% guanine-cytosine and 59% adenine-thymine), was dissolved in an 0.1 M NaCl solution in water. In this solution-phase sample (thickness d≈7.5 *μ*m) with ${\text{Na}\;}^{+}$ counterions, the DNA helices are fully hydrated with a water content of 150 water molecules per base pair. A reduced hydration level was implemented in thin DNA films, prepared by exchanging the ${\text{Na}\;}^{+}$ counterions against cetyltrimethylammonium (CTMA) with the procedure reported in Ref. 19. The films of approximately 25 *μ*m thickness were cast on BaF_2_ substrates and placed inside a home-made humidity cell.^20^ The humidity level in the cell and, thus, the hydration level were maintained fixed by connecting the cell to a reservoir containing either a saturated solution of NaBrO_3_ in water or a P_5_O_5_ powder. The corresponding humidity levels are 92% r.h., corresponding to a water content of 20–30 water molecules per base pair, i.e., roughly two closed water layers around the DNA helices, and 0% r.h. with less than 2 water molecules per base pair.^21^

Electric fields imposed by the thermally fluctuating solvent are simulated by the procedure described in detail in Ref. 10. In brief, molecular dynamics simulations were performed with the Gromacs program package^22^ employing cubic boundary conditions and the TIP5P water model.^23^ Electrostatic potential derived partial charges of dimethylphosphate (CH_3_O)_2_${\text{PO}}_{2}^{-}$ (DMP) were taken from Ref. 24, and force field parameters are taken from the CHARMM27 force field^25^ of DNA. Intramolecular degrees of freedom of DMP were restricted with the SHAKE algorithm. Equilibration was performed as described in Ref. 26; the first 100 ps of the 1 ns production run were further used for equilibration. We consider four equilibrated trajectories of the *gauche-gauche* (gg) conformer of DMP at temperatures T = 298, 310, 320, and 330 K. The fluctuating electric field is evaluated up to quadrupoles within the framework of the Effective Fragment Potential (EFP) model^27^ with a homewritten C++ code linked to the *libefp* library.^28^ Electric fields are recorded at the midpoint of the oxygen-oxygen axis upon projection on the C_2_ symmetry axis of the (PO_2_)^−^ group and averaged over 450 ps for each trajectory.

## III. RESULTS

### A. Linear infrared absorption spectra

**FIG. 1.**
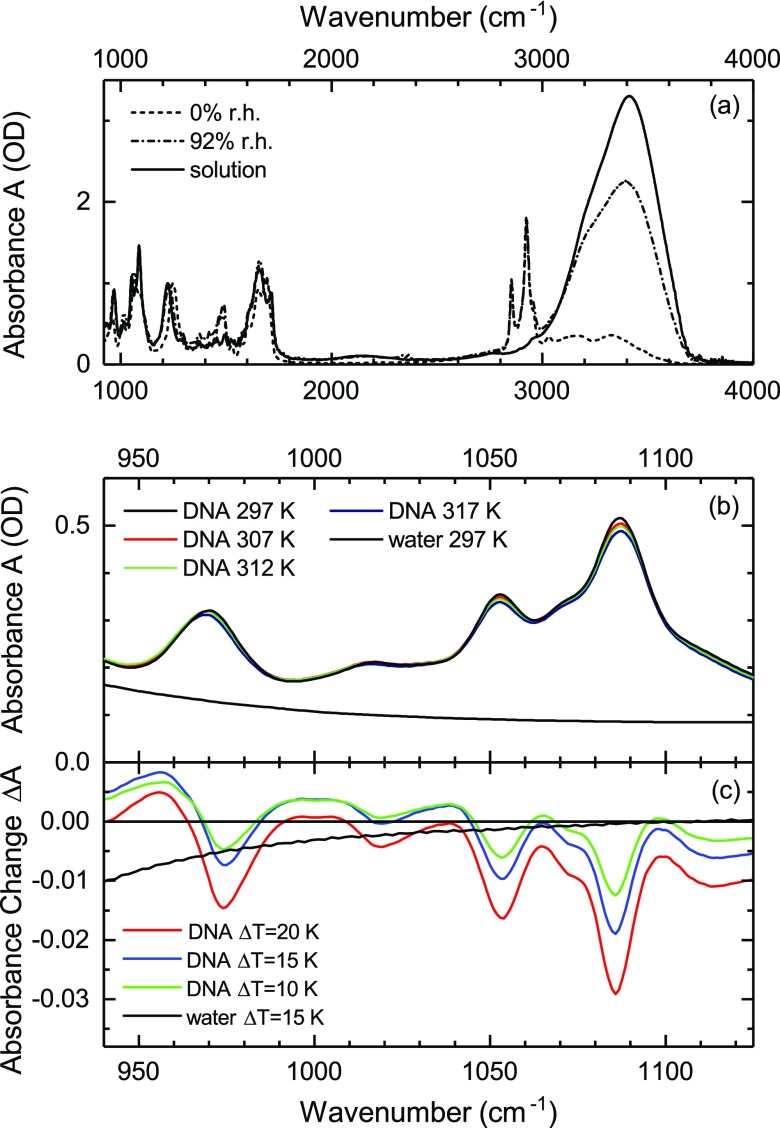
(a) Linear infrared absorption spectra of salmon testes DNA at different hydration levels. The spectra at 0% (less than 2 water molecules per base pair) and 92% r.h. (20–30 water molecules per base pair) were measured with DNA films in which the Na^+^ counterions were replaced by ionic CTMA. The solution-phase sample contains DNA with Na^+^ counterions. (b) Infrared absorption of DNA backbone modes measured with the solution phase sample at different temperatures. The black line gives the contribution of water to the overall infrared absorption. (c) Difference spectra of DNA in solution for different temperature changes ΔT relative to ambient temperature (297 K). The difference spectrum of bulk water is shown for ΔT = 15 K.

Linear infrared absorption spectra of the three DNA samples are summarized in Fig. 1(a) where the absorbance A=−log(T) (*T*: sample transmission) is plotted as a function of wavenumber (cm^−1^). For the two hydrated samples (solution and 92% r.h.), the absorption between 3000 and 4000 cm^−1^ is dominated by the OH stretch band of the water shell. In contrast, the different NH stretch bands of the base pairs prevail in the dry sample (0% r.h.) with a minor contribution from residual water molecules.^29^ The group of narrow absorption bands close to 3000 cm^−1^ is due to CH stretch transitions of DNA and—in the thin-film samples—of CTMA counterions. The fingerprint range below 2000 cm^−1^ includes the OH bend vibration of water and DNA fingerprint modes.

In Fig. 1(b), the absorption spectrum of backbone vibrations of solution-phase DNA is shown in the range from 940 to 1150 cm^−1^ for different sample temperatures T. There are six backbone normal modes which contribute to the spectrum, the symmetric stretch mode of the PO_2_ unit (P2), the diester linker modes L1–L3, and the deoxyribose ring modes R1 and R2 (Table I, Refs. 30 and 31). The normal modes include elongations of different local bonds, i.e., are partially delocalized along a backbone segment between two phosphate groups. The surrounding water gives rise to a broad absorption background (black solid line in Fig. 1(b), T = 297 K) which originates from high-frequency librations of water molecules.

Fig. 1(c) displays difference absorption spectra of the DNA sample for a temperature increase of ΔT = 10, 15, and 20 K relative to ambient temperature (T = 297 K). In addition, a difference spectrum of neat water is shown for ΔT = 15 K. Together with a decrease of the peak absorbance, the spectral envelopes undergo a moderate reshaping with increasing temperature. For the maximum ΔT = 20 K, the relative changes of absorbance at the different maxima are on the order of 5%, significantly larger than the density change of the water solvent. The data are not corrected for the thermal expansion of the CaF_2_ cell windows which would result in a relative change of the sample thickness of less than 10^−2^. It should be noted that the DNA difference spectra contain contributions from both DNA itself and the water shell.

### B. Direct excitation of backbone modes

**FIG. 2.**
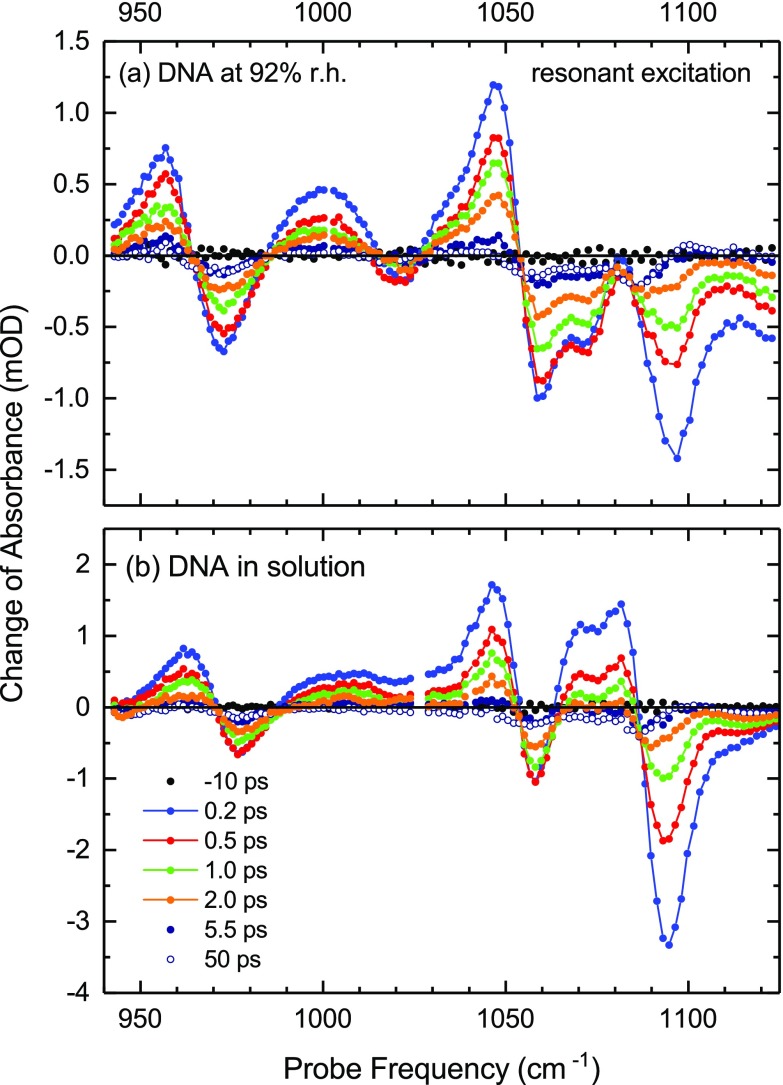
Transient pump-probe spectra of (a) DNA at 92% r.h. and (b) DNA in aqueous solution. The backbone modes are directly excited by the pump pulses. The change of absorbance ΔA = $-\text{log}(T/T_{0})$ in mOD is shown as a function of probe frequency for pump-probe delays up to 50 ps (*T*, *T*_0_: sample transmission with and without excitation). Positive signals ΔA>0 are due to v = 1 to 2 transitions of the vibrations while negative signals ΔA<0 occur on v = 0 to 1 transitions. The weak dispersive lineshapes at late delay times point to small spectral shifts of the v = 0 to 1 transitions in the heated vibrational manifold of DNA.

Pump-probe measurements with direct excitation of the backbone modes were performed in order to determine the vibrational lifetimes and characterize vibrational relaxation of DNA embedded in a water shell in its equilibrium ground state. In Fig. 2, two sets of transient spectra are presented for (a) DNA at 92% r.h. and (b) DNA in solution. The change of absorbance ΔA = $-\text{log}(T/T_{0})$ is plotted as a function of probe frequency for different delay times between the pump and the probe (*T*, *T*_0_: sample transmission with and without excitation). At all spectral positions, the absorption changes rise within the 100 fs time resolution of the experiment. Positive absorption changes are due to the v = 1 to 2 transitions from the transiently populated v = 1 states while negative absorption changes reflect the pump-induced bleaching of the v = 0 ground state and stimulated emission on the v = 1 to 0 transitions. With increasing hydration, the absorption changes at 1090 cm^−1^ with a predominant contribution from the symmetric ${\text{PO}}_{2}^{-}$ stretch vibration become significantly stronger. The enhanced v = 1 to 2 absorption of this mode causes a sign change of the transient spectra in the range between 1065 and 1085 cm^−1^. Here, the net signal represents the difference of this v = 1 to 2 absorption and the bleaching of the v = 0 to 1 transitions of the diester linker modes L1 and L2 at 1050 and 1070 cm^−1^.

The changes of vibrational absorption decay by depopulation of the v = 1 states within some 5 ps to small residual signals. A numerical analysis of decay kinetics of the different bleaching signals (not shown) gives the monoexponential decay times summarized in Table I. Such numbers are considered more reliable than decay times of enhanced absorption which—due to the small frequency separation of the different bands—is influenced by both v = 1 population kinetics and the reshaping of adjacent absorption bands. As has been discussed in Ref. 14, the few-picosecond decay times are determined by both vibrational relaxation of the v = 1 back to the v = 0 ground state of the respective mode and vibrational energy transfer from backbone modes at higher to those at lower frequencies. The small residual absorption changes which occur in the range of the different v = 0 to 1 transitions and persist up to delay times beyond 100 ps reflect spectral shifts of the bands in the heated vibrational manifold of the DNA.

**TABLE I.** DNA backbone vibrations. The frequency positions are taken from the linear infrared absorption spectra. The $\nu_{R2}$ and $\nu_{L3}$ modes contribute to a single absorption band with the maximum position given below. The decay times *τ* are derived from the bleaching components in the pump-probe data measured with direct backbone excitation.

Mode	Character	Frequency at 92% r.h. (cm^−1^)	Frequency in solution (cm^−1^)	*τ* at 92% r.h. (ps)	*τ* in solution (ps)
$\nu_{P2}$	Symmetric phosphate stretch	1089	1087	1.2 ± 0.2	1.0 ± 0.2
$\nu_{L1}$	Diester linkage	1065	1069	1.6 ± 0.2	…
$\nu_{L2}$	Diester linkage	1058	1052	1.6 ± 0.2	2.3 ± 0.3
$\nu_{R1}$	Furanose ring	1014	1017	…	…
$\nu_{R2}$	Ribose main chain	966	968	1.5 ± 0.2	1.7 ± 0.2
$\nu_{L3}$	Diester linkage				

### C. Backbone response after water excitation

**FIG. 3.**
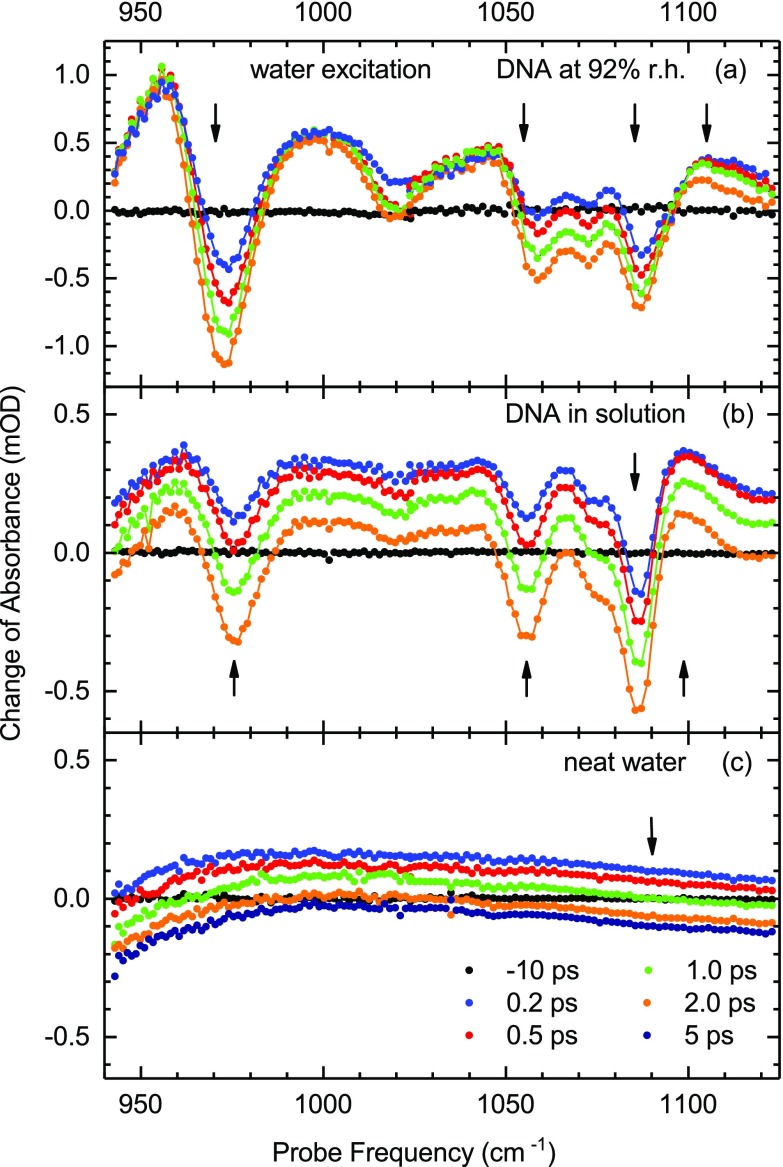
(a) Transient pump-probe spectra of (a) DNA at 92% r.h., (b) DNA in solution, and (c) neat water after excitation of the water OH stretch vibration by pulses centered at 3450 cm^−1^. The change of absorbance $\Delta A=-\text{log}(T/T_{0})$ in mOD is plotted as a function of probe frequency for different delay times (*T*, *T*_0_: transmission of the sample with and without excitation). The arrows mark the spectral positions at which the time-resolved transients of Figs. 5 and 6 were measured.

**FIG. 4.**
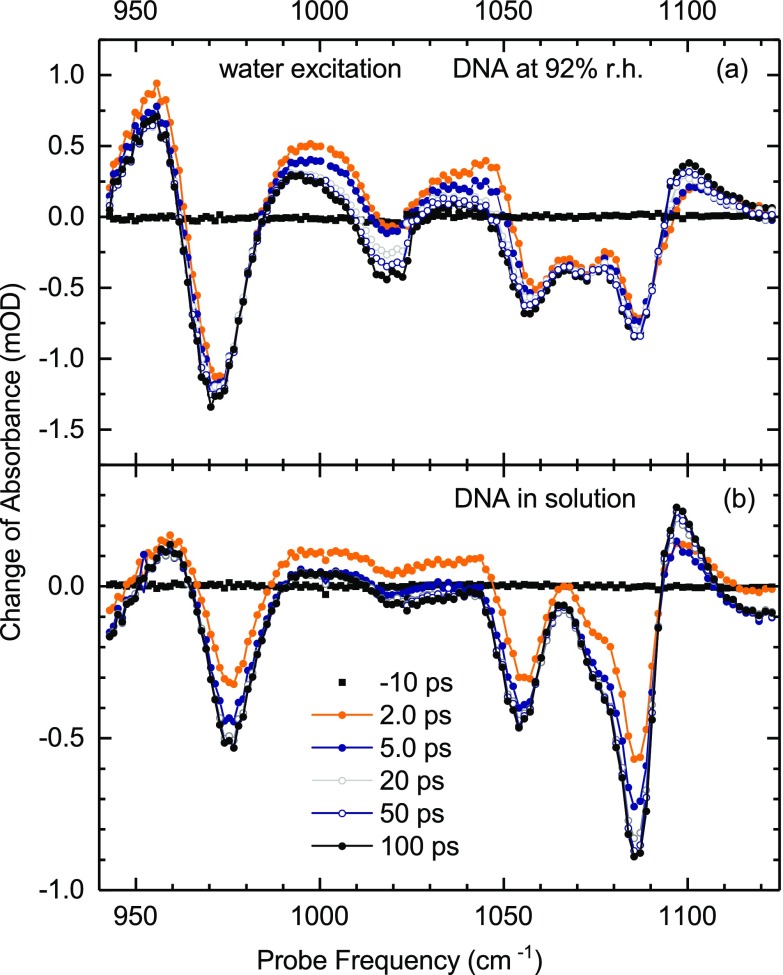
Transient pump-probe spectra of (a) DNA at 92% r.h. and (b) DNA in solution for late delay times between 2 and 100 ps.

An extensive set of data was generated with pumping the OH stretch vibration of water molecules by a pulse centered at 3450 cm^−1^ and probing the response of DNA backbone vibrations. In this scheme, there is no direct excitation of backbone modes to their v = 1 states. In Fig. 3, transient spectra are presented for (a) DNA at 92% r.h., corresponding to roughly two closed water layers around the double helices, (b) DNA in solution, and (c) neat water. The DNA spectra cover a range of delay times up to 2 ps while the water spectra extend to 5 ps. DNA spectra recorded at longer delay times between 2 and 100 ps are summarized in Fig. 4. The transient spectra for 92% r.h. (Fig. 3(a)) are dominated by the DNA response while the spectra of the solution sample consist of contributions from DNA and from the water environment. The latter gives rise to a broadband absorption enhancement at early delay times, similar to what is shown in Fig. 3(c) for neat water.

The transient spectra of Figs. 3(a) and 3(b) display a response of all backbone modes occurring simultaneously with the excitation of the water shell. The backbone response is characterized by a reshaping of spectral envelopes with a pronounced bleaching at the spectral positions of the vibrational bands in the linear absorption spectrum and absorption enhancements in-between these positions. The P2 band at 1087 cm^−1^ displays a blueshift giving rise to the enhanced absorption above 1100 cm^−1^. During the first two picoseconds subsequent to water excitation, the bleaching components become more intense while the reshaping of the positive signal components remains limited. In the solution sample, the broadband enhancement of water's librational absorption makes a significant contribution at early delay times and decays to a residual negative band that remains unchanged after 5 ps (cf. Fig. 3(c)).

The DNA spectra undergo a further reshaping on a slower time scale up to 100 ps, as shown in Fig. 4. The bleaching components become more pronounced while the amplitudes of the positive absorption changes are slightly reduced, except for the feature around 1100 cm^−1^. The latter increases in strength and becomes spectrally narrower. It is important to note that the DNA response after water excitation is markedly different from what is observed after resonant excitation (cf. Fig. 2). Water excitation induces a bleaching of vibrational absorption which rises continuously up to 100 ps while the signals measured with resonant excitation reach their maxima at early delay times and then decay to small residual values at late delay times.

**FIG. 5.**
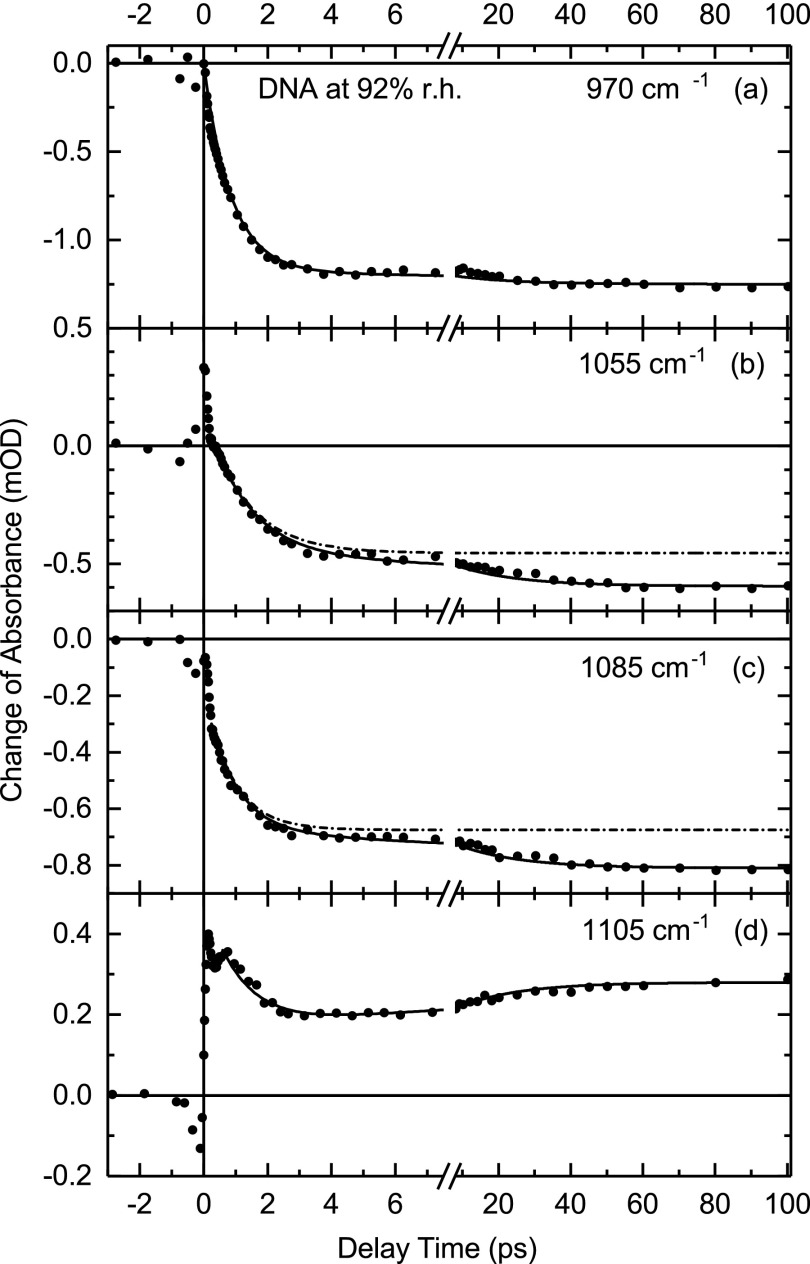
Time resolved pump-probe transients for DNA at 92% r.h. after OH stretch excitation of the water shell (cf. Fig. 3). The absorbance change measured at fixed probe frequencies is plotted as a function of pump-probe delay (symbols). The solid lines are numerical fitting curves to the data, comprised of a fast rise or decay (d) with a time constant between 0.9 and 1.3 ps depending on the particular probe frequency, and a slow rise with 17 ± 4 ps. The dashed-dotted lines in (b) and (c) represent transients calculated without the slow rise.

**FIG. 6.**
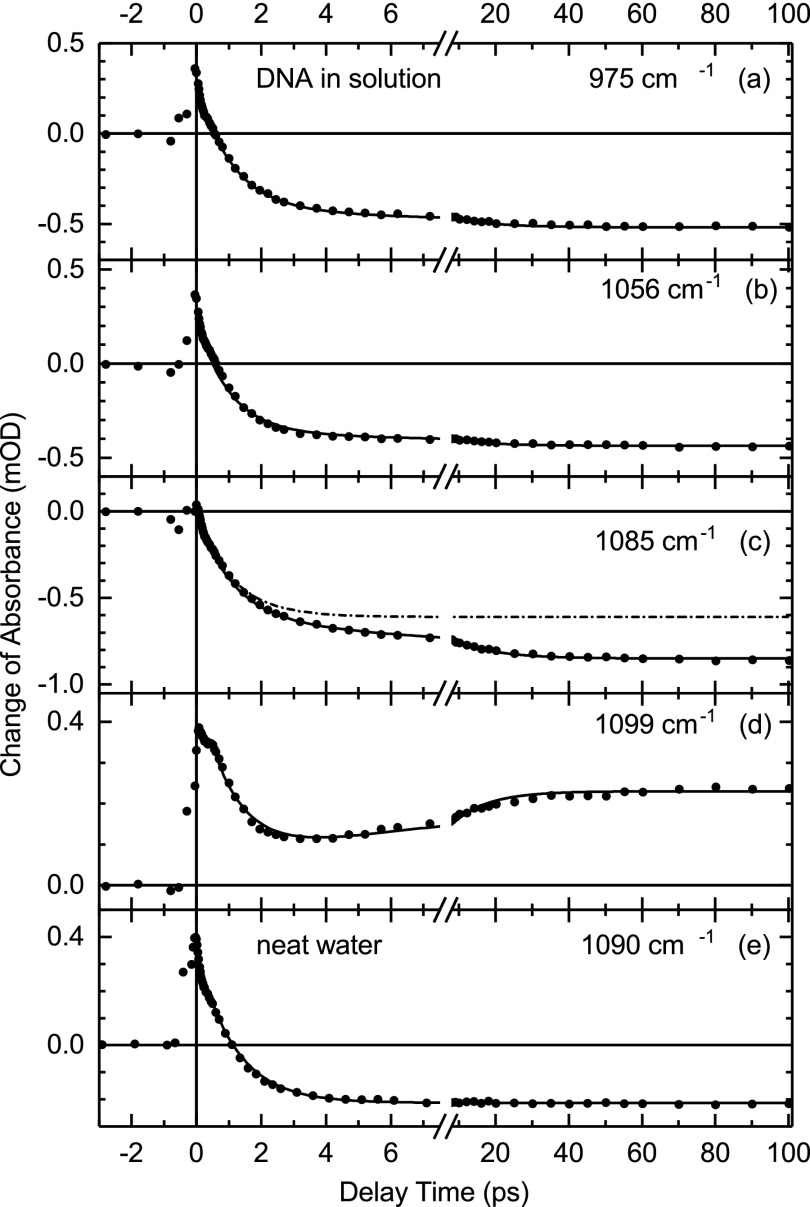
(a)–(d) Time resolved pump-probe transients for DNA in solution recorded at different probe frequencies after OH stretch excitation of the water shell (symbols). (e) Transient absorbance change of neat water after OH stretch excitation. The fitting curves (solid lines) of the DNA data give an initial rise or decay (d) time of 1.1 ± 0.1 ps and a slower rise with 11 ± 2 ps. The water transient is reproduced by an initial 1.1 ps decay to the residual negative change of absorbance.

Time resolved absorption traces recorded at fixed probe frequencies (arrows in Fig. 3) are presented in Fig. 5 for DNA at 92% r.h. and in Fig. 6 for DNA in solution. For comparison, the neat water response is included in Fig. 6(e). The DNA transients at 1105 cm^−1^ (Fig. 5(d)) and 1099 cm^−1^ (Fig. 6(d)) show an initial decay with a time constant of 1 ps followed by a slow rise (solid lines). All other DNA transients show an initial rise within the first 4 ps, followed by a slower rise of smaller amplitude within tens of picoseconds. These kinetics are accounted for by a biphasic exponential rise of the absorption changes (solid lines). For DNA at 92% r.h., one derives time constants between 0.9 and 1.3 ps for the fast rise, depending on the spectral positions, and 17 ± 4 ps for the slow rise at all spectral positions. The dashed-dotted lines in Figs. 5(b) and 5(c) represent kinetics calculated with the fast rise only. DNA in solution displays a fast rise with 1.1 ± 0.1 ps and a slow rise with 11 ± 2 ps at all spectral positions. The neat water transient is reproduced by a 1.1 ps decay to the negative residual absorption change, i.e., there is no slow component.

## IV. DISCUSSION

The pump-probe data recorded with direct excitation of the backbone give insight into population kinetics of the v = 1 states of the different backbone modes. The v = 1 populations originate from both direct excitation by the pump pulse and population transfer from excited backbone modes at higher frequencies.^14^ The latter mechanism leads to a delayed proliferation of v = 1 population with a time constant on the order of 2 to 3 ps, thus lengthening the population decay. The overall decay times of the v = 1 states derived from the pump-probe data are in a range from 1.0 to 2.5 ps (cf. Table I). The v = 1 relaxation represents the first step of energy redistribution within DNA, followed by relaxation of the accepting modes and redistribution within the vibrational manifold of DNA. To form a vibrationally heated ground state, spatial transport of excess energy from the excited backbone parts along the double helix is required. Current insight into the spatial spreading of excess energy in DNA is very limited whereas a typical time scale between 5 ps and tens of picoseconds has been determined for chain-like hydrocarbons and for peptide structures.^32,33^ Given the prominent role of delocalized low-frequency backbone modes in energy transport, its time scales in peptides and DNA should be similar. The introduction of vibrational marker groups in DNA, in analogy to Ref. 33, may allow for more detailed studies of energy transport along the double helix. In parallel to intra-DNA transport, part of the excess energy is expected to be transferred to the water shell, in particular through the hydrated phosphate groups of the backbone. The small long-lived frequency shifts observed in the pump-probe spectra at late delay times are a hallmark of the heated ground state in which low-frequency modes coupling to backbone vibrations display a thermally enhanced population, thus inducing the spectral shifts.^34^

We now discuss the processes occurring after excitation of the water shell via the OH stretch vibration. Extensive experimental and theoretical work on neat water has provided detailed quantitative insight into this relaxation scenario.^35^ The OH stretch vibration of H_2_O has a lifetime of approximately 200 fs and decays via the v = 2 and v = 1 states of the OH bend vibration.^36^ The v = 1 lifetime of the OH bending mode is 170 fs.^37^ In the first step from the v = 2 to the v = 1 state of the OH bend vibration, an amount of energy equivalent to an OH bend quantum is transferred to librational degrees of freedom, followed by a similar transfer in the relaxation from the v = 1 to the v = 0 state of the bending vibration. According to recent theoretical work, the main accepting libration is the L2 libration of the bend-excited water molecules.^38,39^ From this degree of freedom, energy is very rapidly (<100 fs) transferred into the manifold of intermolecular modes and a hot vibrational ground state of the water shell is formed with a time constant of approximately 1 ps.

In the present experiments, the water concentration in the DNA sample at 92% r.h. and the solution-phase sample is 10 M and 44 M, respectively. The resulting optical penetration depths of the pump pulses are 10.5 and 2.4 *μ*m with a fraction of 3 to 4% of excited water molecules in the irradiated sample volume. The spatial distribution of excited water molecules is distinctly different in the two cases: in the sample at 92% r.h. with 20–30 water molecules per DNA base pair, all excited water molecules are arranged in the two water layers around the DNA double helix, i.e., in direct proximity. In contrast, 80% of the water molecules excited in the DNA solution sample with 150 waters per base pair are beyond the first two layers. From the pump energy deposited in the respective sample volume and the specific heat of water, one estimates a temperature increase in the final hot water ground state of some 22 K.

The DNA response after excitation of the water shell displays an initial rise time of 1.1 ps and a subsequent slower rise with time constants of 17 ps for DNA at 92% r.h. and 11 ps for DNA in solution. The rise time of the first component is identical to the formation time of the hot ground state in the water shell. We conclude from this key result that (i) the DNA backbone vibrations remain in their v = 0 states during water relaxation and (ii) the observed absorption changes represent changes of the spectral envelopes of the different v = 0 to 1 absorption bands. A direct energy transfer from water's OH stretch and/or bend vibrations to the DNA backbone modes, e.g., by dipole-dipole coupling, can safely be excluded because of the large energy mismatch between the transitions. Moreover, any transient population of v = 1 states of DNA modes by water relaxation would introduce additional kinetic components with time constants similar to the decay times measured after direct excitation of the backbone (cf. Table I). As a result, the initial rise of the backbone response after water excitation would be slower than the formation of the hot water ground state, in disagreement with our data.

A central issue is the coupling mechanisms between the water shell and the DNA backbone which make the DNA vibrational lineshapes sensitive to heating of the water environment. A first candidate is intermolecular energy transfer from water into DNA via anharmonically coupled low-frequency modes, in particular through backbone units forming intermolecular hydrogen bonds with the water shell. To be consistent with the observation of identical 1.1 ps rise times of the hot water ground state and the spectral reshaping of *all* DNA vibrational bands, such a transfer would have to occur with time constants much shorter than 1 ps. To account for the fact that all backbone vibrations display the same 1.1 ps rise, independent of the local hydration geometry of the relevant functional groups, also the intra-DNA energy redistribution would have to occur much faster than 1 ps. In view of the existing knowledge on intermolecular energy transfer to and from large polyatomic molecules which suggests energy transfer times between a few and several tens of picoseconds^17,40^ such a scenario is considered highly unrealistic.

The DNA backbone is subject to strong and fluctuating electric fields which mainly originate from water molecules in the first two layers around the double helix. Very recent experimental and theoretical work has shown that the electric field amplitudes reach values as high as 90 MV/cm with fluctuation amplitudes of some 25 MV/cm.^9,10^ The fluctuations are due to the fast thermal motions of water molecules in the water equilibrium structure, with frequencies up to several hundred wavenumbers (cm^−1^). Excitation of the OH stretch and bend modes of water molecules in the neighborhood of the DNA backbone and the subsequent formation of the hot ground state change the spatial arrangement of water molecules and, thus, the electric field acting on the backbone vibrations. To be more specific, population of the v = 1 state of the OH stretch oscillator is expected to induce a strengthening, i.e., shortening of hydrogen bonds around the excited water molecules and a concomitant change of the hydrogen-bonded water network.^41,42^ In contrast, heating of the water network by delocalizing excess energy within a period set by the 1.1 ps relaxation time results in a weakening of hydrogen bonds and an increase in the fraction of less than fourfold coordinated water molecules.^36^ The resulting change of electronic polarization of the backbone affects the potential energy surfaces of the backbone vibrations quasi-instantaneously and gives rise to spectral shifts of vibrational transitions and a reshaping of vibrational bands. In their v = 0 ground state, the backbone modes respond to changes of the vibrational potential within a fraction of their vibrational period, i.e., for the modes considered here within less than 50 fs. Thus, the Coulomb coupling between the rearranging water shell and the backbone vibrations maps the formation of the hot water ground states onto the backbone's vibrational spectra and results in identical kinetics of the water shell and the backbone absorption changes. For the directly hydrated phosphate groups, there may be a minor additional contribution from a thermally induced weakening of hydrogen bonds between water molecules and the PO_2_^−^ groups but electric interactions will play the dominate role.^26,43^

This picture is further supported by the larger amplitudes of the DNA absorption changes which are observed at early delay times in the 92% r.h. sample compared to the solution sample. In the first case, all excited water molecules are located in the first two water layers which generate the relevant electric field while only 20% of water molecules excited in the solution phase sample are part of the first two layers. In view of the short-range character of the electric field at the DNA-water interface,^8,10^ larger changes in the acting electric field and, thus, stronger initial absorption changes occur at the lower water level.

To assess the changes of electric field upon heating of the water shell in a more quantitative way, we performed theoretical calculations on the model system dimethylphosphate (DMP) in water. DMP has been used repeatedly as a model system for the DNA backbone in order to determine its vibrational normal modes and to derive force fields for phosphate vibrations. The phosphate group of DMP is the primary interaction site with water, being hydrated by up to 6 water molecules which form hydrogen bonds with the ${\text{PO}}_{2}^{-}$ oxygens. The present calculations treat the DMP/water system in its equilibrium ground state at different temperatures. Water dynamics are simulated in the molecular dynamics approach described in Sec. II and the resulting distribution of electric field amplitudes at the phosphate group of DMP is derived for different temperatures of the ensemble.

**FIG. 7.**
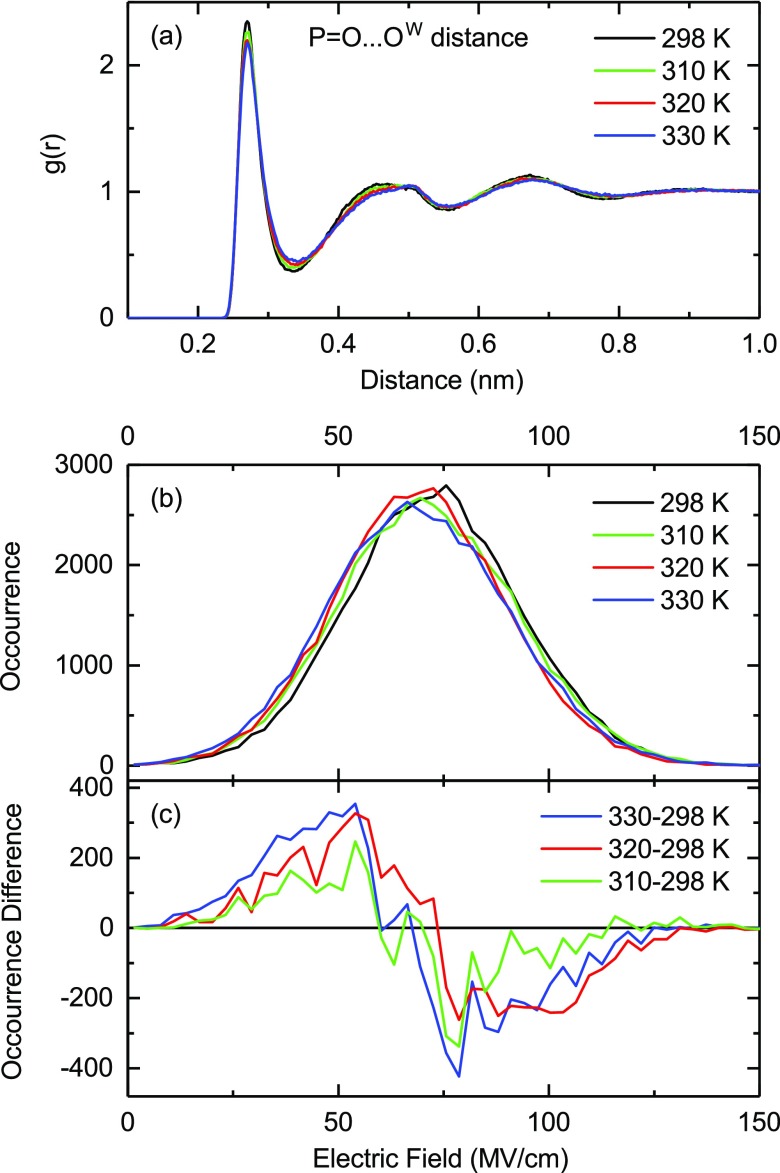
(a) Radial distribution functions g(r) of water molecules as a function of distance between the PO_2_ oxygens of dimethylphosphate and the water oxygens for four different sample temperatures. (b) Time averaged distribution of water shell imposed electric fields projected on the C_2_ symmetry axis of the PO_2_ unit at the midpoint of the oxygen-oxygen axis of DMP for the four different sample temperatures. (c) Occurrence difference of electric fields.

With increasing temperature, the time averaged radial distribution functions g(r) (Fig. 7(a)) display a decrease of water occupancy in the first layer and a concomitant increase and broadening in the second layer. While the spatial rearrangements are moderate, they nevertheless result in a decrease of the electric field acting on the DMP phosphate group. Fig. 7(b) shows the time-averaged distribution of electric field amplitudes at the phosphate group and Fig. 7(c) the differential change, where the differential maximum and minimum are separated by about 25 MV/cm. The reduction of the field amplitudes induces a blueshift of the symmetric and asymmetric PO_2_ stretch vibrations. For the symmetric PO_2_ stretch vibration, one derives a value of Δν=a·ΔE≈10 cm^−1^ with *a* = 0.4 cm^−1^/(MV/cm) taken from the ab-initio calculations of Ref. 43 and ΔE = 25 MV/cm. This value is in good agreement with the differential signal of the symmetric PO_2_ stretch band in the transient DNA spectra at late delay times (Fig. 4(b)) where the bleaching component and absorption enhancement are separated by about 10 cm ^− 1^. We note that mean values of time-averaged electric field amplitudes are only moderately affected for increasing temperature (≈ 5 MV/cm, cf. Fig. 7(b)) corresponding to an absolute spectral shift of the v = 0 to 1 transition of the symmetric PO_2_ stretch vibration of ≈ 2 cm^−1^. An earlier study of artificial DNA oligomers at 92% r.h. has revealed a separation of the bleaching and absorption enhancement component of the asymmetric PO_2_ stretch vibration by some 20 cm^−1^ after OH stretch excitation of the water shell.^44^ This separation which also builds up with the formation time of the hot water ground state is larger than for the symmetric PO_2_ stretch vibration, in line with results from theory.^10,43^ Currently, there are no reliable force fields for the other backbone vibrations and, at present, their smaller frequency shifts cannot be directly quantified.

We finally return to the issue of energy transfer from the heated water shell to DNA. The transients in Figs. 5 and 6 consistently display slower kinetics on a time scale of tens of picoseconds. The transient spectra of Fig. 4 demonstrate that such kinetics are connected with a moderate reshaping of the spectral envelopes. In particular, the symmetric PO_2_ stretching band shows a spectral sharpening. We attribute such changes to a heating of the DNA double helix by energy transfer from the water shell and energy redistribution within the helix on the same time scale of several tens of picoseconds. The quasi-continuous density of states of the vibrational manifold of the DNA helix represents an additional heat sink for the excess energy initially deposited in the water shell, leading to a reduction of the temperature jump in the equilibrated system compared to the initial ΔT≈20 K of the water shell. The absorption changes persistent after equilibration between the water shell and DNA are due to both changes in the electric field the water shell exerts on the backbone and anharmonic couplings of thermally populated low-frequency modes of the double helix to the backbone vibrations. The fact that the late-delay spectra after water excitation are markedly different from the late delay spectra after direct DNA excitation (cf. Fig. 2), the latter showing much smaller absorption changes, may indicate that the field-induced changes predominate over effects originating from anharmonic coupling.

The pump-probe spectra of the solution-phase DNA sample measured at late delay times (Fig. 4(b)) bear out spectral features very similar to the stationary difference spectra of Fig. 1(c) which were derived from the linear absorption spectra recorded at different temperatures, i.e., under conditions where DNA and water shell are at the same temperature. There is, however, one major difference between the steady-state and the pump-probe measurements: In the steady-state case, heating is connected with a thermal change of the macroscopic mass density in the sample, affecting the molecular arrangements in the water shell and—to lesser extent—in the DNA. After ultrafast excitation, the macroscopic density change builds up by acoustic phonon propagation in the sample, well beyond the time range covered in the pump-probe experiments. The transient DNA spectra for time delays around 100 ps thus reflect conditions under which the excess energy has been equilibrated between water shell and DNA but the mass density is still at a value characteristic for the initial lower temperature.

## V. CONCLUSIONS

In conclusion, we have presented a detailed femtosecond infrared study of interactions between DNA and its hydration shell at two different hydration levels. Using backbone vibrations as sensitive probes of molecular couplings and energy exchange at and through the DNA-water interface, the response of the backbone to excitation of the water shell was mapped in a temporally and spectrally resolved way. The backbone vibrations display a biphasic response with a fast 1 ps component occurring in parallel to the formation of a hot ground state in the water shell and a subsequent slower contribution developing with time constants between 10 and 20 ps. The observed changes of the DNA vibrational spectra are due to a reshaping of the v = 0 to 1 absorption bands of the different backbone modes which stay in their v = 0 ground state under the present experimental conditions. Coupling between the water shell and the backbone is predominantly mediated by the electric field the water shell exerts on the backbone. The electric field distribution changes upon formation of the hot water ground state which is connected with a limited relocation of water molecules in the first and second layers around the double helix. The fast component of the DNA response is due to this Coulomb-mediated coupling and the observed blue-shift of the symmetric PO_2_ stretching band is in agreement with estimates based on MD simulations of the change in electric field distribution and the relevant force field. Energy transfer into the DNA double helix occurs on a slower time scale of tens of picoseconds and establishes a common heated ground state of water shell and DNA. The redistribution of excess energy within the DNA structure requires energy delocalization throughout the double helix, a process occurring on a similar time scale of tens of picoseconds. Further studies are required to clarify the kinetics and mechanisms of the latter two processes in more detail.

Our results demonstrate the pronounced sensitivity of biomolecular vibrations and their absorption spectra to electric fields exerted by an aqueous environment. The underlying Coulomb couplings are comparably strong and—in the present case—seem to prevail over anharmonic couplings and their impact on the vibrational spectra. Future work will need to address the Coulomb couplings in a more quantitative way for which studies of model systems with well-defined geometries and water content as well as the application of electric field transients in the terahertz range will be as important as in-depth theoretical calculations and simulations. Another open issue is the molecular pathways of energy transfer between biomolecules and their water shells.

## References

[1] Y. Levy and J. N. Onuchic , “ Water mediation in protein folding and molecular recognition,” Annu. Rev. Biophys. Biomol. Struct. , 389–415 (2006). 10.1146/annurev.biophys.35.040405.102134 16689642

[2] W. Saenger , *Principles of Nucleic Acid Structure* ( Springer, Berlin 1984), Chap. 17.

[3] D. Vlieghe , J. P. Turkenburg , and L. van Meervelt , “ B-DNA at atomic resolution reveals extended hydration patterns,” Acta Crystallogr. D , 1495–1502 (1999). 10.1107/S0907444999007933 10489444

[4] M. Egli *et al.*, “ X-ray crystallographic analysis of the hydration of A- and B-form DNA at atomic resolution,” Biopolymers , 234–252 (1998). 10.1002/(SICI)1097-0282(1998)48:4<234::AID-BIP4>3.0.CO;2-H 10699842

[5] M. L. Kopka , A. V. Fratini , H. R. Drew , and R. E. Dickerson , “ Ordered water structure around a B-DNA dodecamer. A quantitative study,” J. Mol. Biol. , 129–146 (1983). 10.1016/0022-2836(83)90033-5 6834428

[6] M. Feig and B. M. Pettitt , “ Modeling high-resolution hydration patterns in correlation with DNA sequence and conformation,” J. Mol. Biol. , 1075–1095 (1999). 10.1006/jmbi.1998.2486 10047483

[7] B. Schneider , K. Patel , and H. M. Berman , “ Hydration of the phosphate group in double-helical DNA,” Biophys. J. , 2422–2434 (1998). 10.1016/S0006-3495(98)77686-6 9788937 PMC1299916

[8] D. J. Floisand and S. A. Corcelli , “ Computational study of phosphate vibrations as reporters of DNA hydration,” J. Phys. Chem. Lett. , 4012–4017 (2015). 10.1021/acs.jpclett.5b01973 26722770

[9] T. Siebert , B. Guchhait , Y. Liu , B. P. Fingerhut , and T. Elsaesser , “ Range, magnitude, and ultrafast dynamics of electric fields at the hydrated DNA surface,” J. Phys. Chem. Lett. , 3131–3136 (2016). 10.1021/acs.jpclett.6b01369 27468144

[10] B. P. Fingerhut , R. Costard , and T. Elsaesser , “ Predominance of short range Coulomb forces in phosphate-water interactions—A theoretical analysis,” J. Chem. Phys , 115101 (2016). 10.1063/1.496275527004898

[11] K. E. Furse and S. A. Corcelli , “ The dynamics of water at DNA interfaces: Computational studies of Hoechst 33258 bound to DNA,” J. Am. Chem. Soc. , 13103–13109 (2008). 10.1021/ja803728g 18767841

[12] M. Yang , Ł. Szyc , and T. Elsaesser , “ Decelerated water dynamics and vibrational couplings of hydrated DNA mapped by two-dimensional infrared spectroscopy,” J. Phys. Chem. B , 13093–13100 (2011). 10.1021/jp208166w 21972952

[13] E. Duboue-Dijon , A. C. Fogarty , J. T. Hynes , and D. Laage , “ Dynamical disorder in the DNA hydration shell,” J. Am. Chem. Soc. , 7610–7620 (2016). 10.1021/jacs.6b02715 27240107

[14] T. Siebert , B. Guchhait , Y. Liu , R. Costard , and T. Elsaesser , “ Anharmonic backbone vibrations in ultrafast processes at the DNA-water interface,” J. Phys. Chem. B , 9670–9877 (2015) (supporting information). 10.1021/acs.jpcb.5b04499 26125542

[15] B. Guchhait , Y. Liu , T. Siebert , and T. Elsaesser , “ Ultrafast vibrational dynamics of the DNA backbone at different hydration levels mapped by two-dimensional infrared spectroscopy,” Struct. Dyn. , 043202 (2016). 10.1063/1.4936567 26798841 PMC4720115

[16] A. C. Fogarty , E. Duboue-Dijon , F. Sterpone , J. T. Hynes , and D. Laage , “ Biomolecular hydration dynamics: A jump model perspective,” Chem. Soc. Rev. , 5672–5683 (2013). 10.1039/c3cs60091b 23612685

[17] T. Elsaesser and W. Kaiser , “ Vibrational and vibronic relaxation of large polyatomic molecules in liquids,” Annu. Rev. Phys. Chem. , 83–107 (1991). 10.1146/annurev.pc.42.100191.000503

[18] R. A. Kaindl , M. Wurm , K. Reimann , P. Hamm , A. M. Weiner , and M. Woerner , “ Generation, shaping, and characterization of intense femtosecond pulses tunable from 3 to 20 *μ*m,” J. Opt. Soc. Am. B , 2086–2094 (2000). 10.1364/JOSAB.17.002086

[19] K. Tanaka and Y. Okahata , “ A DNA-lipid complex in organic media and formation of an aligned cast film,” J. Am. Chem. Soc. , 10679–10683 (1996). 10.1021/ja9617855

[20] J. R. Dwyer , Ł. Szyc , E. T. J. Nibbering , and T. Elsaesser , “ Note: An environmental cell for transient spectroscopy on solid samples in controlled atmospheres,” Rev. Sci. Instrum. , 036101 (2013). 10.1063/1.4794092 23556853

[21] M. Falk , K. A. Hartman , and R. C. Lord , “ Hydration of DNA. II. An infrared study,” J. Am. Chem. Soc. , 387–391 (1963). 10.1021/ja00887a004

[22] B. Hess , C. Kutzner , D. van der Spoel , and E. Lindahl , “ GROMACS 4: Algorithms for highly efficient, load-balanced, and scalable molecular simulation,” J. Chem. Theory Comput. , 435–447 (2008). 10.1021/ct700301q 26620784

[23] M. W. Mahoney and W. L. Jorgensen , “ A five-site model for liquid water and the reproduction of the density anomaly by rigid, nonpolarizable potential functions,” J. Chem. Phys. , 8910–8922 (2000). 10.1063/1.481505

[24] J. Florián , V. Baumruk , M. Štrajbl , L. Bednárová , and J. Štěpánek , “ IR and Raman Spectra, conformational flexibility, and scaled quantum mechanical force fields of sodium dimethyl phosphate and dimethyl phosphate anion,” J. Phys. Chem. , 1559–1568 (1996). 10.1021/jp9520299

[25] A. D. MacKerell, Jr. , N. Banavali , and N. Foloppe , “ Development and current status of the CHARMM force field for nucleic acids,” Biopolymers , 257–265 (2001). 10.1002/1097-0282(2000)56:4%3C257::AID-BIP10029%3E3.0.CO;2-W 11754339

[26] R. Costard , T. Tyborski , B. P. Fingerhut , and T. Elsaesser , “ Ultrafast phosphate hydration dynamics in bulk H_2_O,” J. Chem. Phys. , 212406 (2015). 10.1063/1.4914152 26049426

[27] P. N. Day , J. H. Jensen , M. S. Gordon , S. P. Webb , W. J. Stevens , M. Krauss , D. Garmer , H. Basch , and D. Cohen , “ An effective fragment method for modeling solvent effects in quantum mechanical calculations,” J. Chem. Phys. , 1968–1986 (1996). 10.1063/1.472045

[28] I. A. Kaliman and L. V. Slipchenko , “ LIBEFP: A new parallel implementation of the effective fragment potential method as a portable software library,” J. Comput. Chem. , 2284–2292 (2013). 10.1002/jcc.23375 24159627

[29] Ł. Szyc , J. R. Dwyer , E. T. J. Nibbering , and T. Elsaesser , “ Ultrafast dynamics of N-H and O-H stretching excitations in hydrated DNA oligomers,” Chem. Phys. , 36–44 (2009). 10.1016/j.chemphys.2008.08.013

[30] Y. Guan and G. Thomas , “ Vibrational analysis of nucleic acids. IV. Normal modes of the DNA phosphodiester structure modeled by diethyl phosphate,” Biopolymers , 813–835 (1996). 10.1002/(SICI)1097-0282(199612)39:6<813::AID-BIP7>3.3.CO;2-C 8946802

[31] M. Banyay , M. Sarkar , and A. Gräslund , “ A library of IR bands of nucleic acids in solution,” Biophys. Chem. , 477–488 (2003). 10.1016/S0301-4622(03)00035-8 12878315

[32] Z. Wang , J. A. Carter , A. Lagutchev , Y. K. Koh , N.-H. Seong , D. G. Cahill , and D. D. Dlott , “ Ultrafast flash thermal conductance of molecular chains,” Science , 787–790 (2007). 10.1126/science.1145220 17690290

[33] V. Botan , E. H. G. Backus , R. Pfister , A. Moretto , M. Crisma , C. Toniolo , P. H. Nguyen , G. Stock , and P. Hamm , “ Energy transport in peptide helices,” Proc. Natl. Acad. Sci. U. S. A. , 12749–12754 (2007). 10.1073/pnas.0701762104 17646650 PMC1937538

[34] P. Hamm , S. Ohline , and W. Zinth , “ Vibrational cooling of azobenzene after photoisomerisation measured by femtosecond IR spectroscopy,” J. Chem. Phys. , 519–529 (1997). 10.1063/1.473392

[35] H. J. Bakker and J. L. Skinner , “ Vibrational spectroscopy as a probe of structure and dynamics in liquid water,” Chem. Rev. , 1498–1517 (2010). 10.1021/cr9001879 19916491

[36] S. Ashihara , N. Huse , A. Espagne , E. T. J. Nibbering , and T. Elsaesser , “ Ultrafast structural dynamics of water induced by dissipation of vibrational energy,” J. Phys. Chem. A , 743–746 (2007). 10.1021/jp0676538 17266211

[37] N. Huse , S. Ashihara , E. T. J. Nibbering , and T. Elsaesser , “ Ultrafast vibrational relaxation of O-H bending and librational excitations in liquid H_2_O,” Chem. Phys. Lett. , 389–393 (2005). 10.1016/j.cplett.2005.02.007

[38] R. Rey , F. Ingrosso , T. Elsaesser , and J. T. Hynes , “ Pathways for H_2_O bend vibrational relaxation in liquid water,” J. Phys. Chem. A , 8949–8962 (2009). 10.1021/jp9036342 19719303

[39] R. Rey and J. T. Hynes , “ Solvation dynamics in liquid water. 1. Ultrafast energy fluxes,” J. Phys. Chem. B , 7558–7570 (2015). 10.1021/jp5113922 25635521

[40] C. T. Middleton , K. de La Harpe , C. Su , Y. K. Law , C. E. Crespo-Hernandez , and B. Kohler , “ DNA excited-state dynamics: From single bases to the double helix,” Annu. Rev. Phys. Chem. , 217–239 (2009). 10.1146/annurev.physchem.59.032607.093719 19012538

[41] S. Woutersen and H. J. Bakker , “ Hydrogen bond in liquid water as a Brownian oscillator,” Phys. Rev. Lett. , 2077–2080 (1999). 10.1103/PhysRevLett.83.2077

[42] K. Kwac and E. Geva , “ Mixed quantum-classical molecular dynamics study of the hydroxyl stretch in methanol/carbon-tetrachloride mixtures II: Excited state hydrogen bonding structure and dynamics, infrared emission spectrum, and excited state lifetime,” J. Phys. Chem. B , 2856–2866 (2012). 10.1021/jp211792j 22283660

[43] N. M. Levinson , E. E. Bolte , C. S. Miller , S. A. Corcelli , and S. G. Boxer , “ Phosphate vibrations probe local electric fields and hydration in biomolecules,” J. Am. Chem. Soc. , 13236–13239 (2011). 10.1021/ja2042589 21809829 PMC3161143

[44] Ł. Szyc , M. Yang , and T. Elsaesser , “ Ultrafast dynamics of water-phosphate interactions in hydrated DNA,” J. Phys. Chem. B , 7951–7957 (2010). 10.1021/jp101174q 20481569

